# Sonographic swelling of pronator quadratus muscle in patients with occult bone injury

**DOI:** 10.1186/s12880-015-0051-6

**Published:** 2015-03-15

**Authors:** Junko Sato, Yoshinori Ishii, Hideo Noguchi, Shin-ichi Toyabe

**Affiliations:** Ishii Orthopaedic and Rehabilitation Clinic, 1089 Shimo-Oshi, Gyoda, Saitama 361-0037 Japan; Division of Information Science and Biostatistics, Niigata University Graduate School of Medical and Dental Sciences, 1 Asahimachi Dori, Niigata, Niigata 951-8520 Japan

**Keywords:** Ultrasonography, Pronator quadratus, Occult bone injury, Wrist joint

## Abstract

**Background:**

The disarranged fat stripe of the pronator quadratus muscle (PQ) on radiographs (the PQ sign) is reported to be predictive of subtle bone fractures. This study aimed to report the results of magnetic resonance imaging (MRI) study in the patients in whom bone injury was not radiographically detected around the wrist joint, and the PQ was sonographically swollen following acute trauma.

**Methods:**

We evaluated sonographically the PQ of 55 patients who showed normal radiographs following acute trauma. The sonographic appearance of the PQ was checked on both longitudinal and transverse images. On the longitudinal image, the probe was positioned along the flexor carpi radialis tendon. For the transverse image, we adopted the image of the same level in which the PQ of the unaffected hand showed maximal thickness. The PQ was considered to be swollen with disproportionate hyperechogenicity and/or thickening compared with the unaffected side at least in one of the two images. Of the 55 patients, 25 patients whose PQ was considered to be swollen underwent MRI study. PQ thickness in millimeters was retrospectively measured on longitudinal and transverse sonographic images.

**Results:**

Twenty-three patients (92.0%) had occult bone injury, and two adult patients (8.0%) showed only wrist joint effusion on MRI. Among these 23, the distal radius was the most frequent location of the occult bone injury (20 patients; 9 [36.0%] with an occult fracture line and 11 [44.0%] with bone bruising). In longitudinal image, the mean value of the PQ thickness of affected hands was 6.2 (3.7–9.6 mm; standard deviation [SD], 1.5) and that of unaffected hands was 4.5 (2.3–6.7 mm; SD, 1.2), respectively. In transverse image, that of dominant and nondominant hands was 7.6 (4.6–13.2 mm; SD, 2.0) and 5.5 (3.6–7.5 mm; SD, 1.1), respectively. The mean difference in PQ thickness between affected and unaffected hands was 1.7 (0.1–5.0 mm; SD, 1.1) in longitudinal image and 2.0 (0.3–6.8 mm; SD, 1.7) in transverse image.

**Conclusions:**

Sonographic swelling of the PQ might be indicative of occult bone injury in patients with normal radiographs following acute trauma.

## Background

MacEwan [1] reported the usefulness of the radiolucent plane overlying the pronator quadratus (PQ) seen on the lateral radiograph of the distal forearm in patients with undisplaced fractures of the radius and ulna, namely the “PQ sign”, which is an anterior bowing or obliteration of the stripe of fat plane paralleling between the PQ and flexor digitorum profundus muscle.

The PQ sign is visible when fluid accumulates within the PQ and the muscle becomes swollen, bulging anteriorly. The PQ occupies a distinct space without intermuscular communication in the deeper part of anterior forearm [2], and this anatomical feature seems to contribute to the appearance of the PQ sign. Moosikasuwan [3] speculated that this sign would be false-negative in such situations as the fractured level differs from the level of the PQ, the fascia covering the muscle is torn, or the radiograph is of poor quality.

Despite high sensitivity and specificity for the diagnosis of fracture in the first report of the PQ sign, with values of 98% and 94%, respectively [1], subsequent studies did not always report the clinical importance of this radiographic abnormality. Zammit-Maempel et al. [4] reported only 51% of forearm fractures showed abnormal pronator fat stripe. Annamalai and Raby [5] reported that the sensitivity and specificity of the PQ sign were even less, with values of 26% and 70%, respectively in the radiographically occult fractures detected on follow-up magnetic resonance imaging (MRI). MRI seems to be the most reliable imaging modality in terms of its ability to diagnose occult bone injury; however, it is expensive and time-consuming. On the other hand, ultrasonography has great benefits in its noninvasiveness, low cost, portability and rapidity which make it easier to use in the outpatient clinic and emergency room; however, one major disadvantage of this imaging modality is the inability in qualitative diagnosis of bone marrow.

The purpose of this study was to investigate the usefulness of the sonographic PQ swelling in identifying occult bone injury following acute trauma.

## Methods

Approval for this study was obtained from the institutional ethics committee of Healthcare Corporation Ashinokai, Gyoda, Saitama, Japan, and all patients were informed of study aims and procedures and signed a consent form that included a description of the protocol. In a period of 1 year, 56 consecutive patients who visited our clinic for wrist injury with a history of acute trauma within 7 days and normal initial radiographs underwent sonographic studies. All patients routinely underwent plain radiography of the wrist joint (standard dorsal-volar and lateral views) prior to the sonographic study at the initial visit to our clinic. For patients with apparent tenderness at the anatomical snuff box, we added two radiographs to check for scaphoid fracture: a dorsal-volar view with the wrist in ulnar deviation and with the thumb clenched by the other fingers, and a semipronated oblique view. The decision of additional sonographic studies depended on the presence of pain disproportionate to normal radiographs. Exclusion criteria included rheumatoid arthritis, dialysis treatment, open wounds, or a history of major hand trauma and/or major hand surgery. As a result, 55 patients were enrolled in this study, excluding one pediatric male patient with a history of a distal radius fracture on the contralateral side.

**Figure 1:**
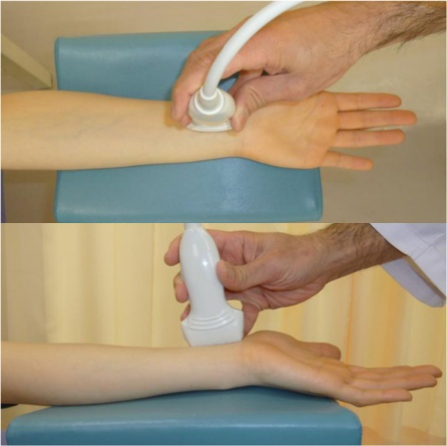
**Position of the probe in the sonographic study on the longitudinal image.**

**Figure 2:**
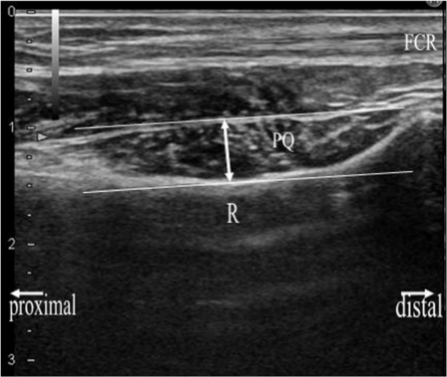
**Sonographic longitudinal image of the examined part of the distal forearm, including anatomical reference and measurements of PQ thickness, using a 17-MHz transducer.** Left hand of a 33-year-old healthy male volunteer. We referred to the line tangential to the volar concave aspect of the radius. FCR: flexor carpi radialis; PQ: pronator quadratus; R: radius; full-lined arrow: maximum thickness of the PQ.

**Figure 3:**
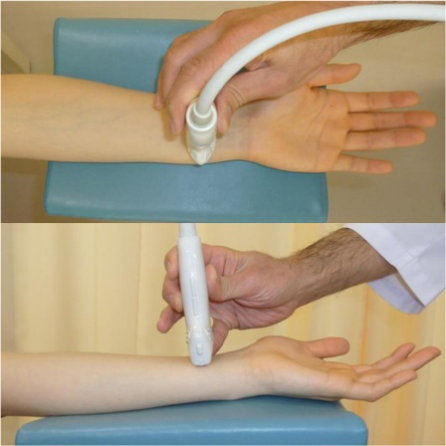
**Position of the probe in the sonographic study on the transverse image.**

**Figure 4:**
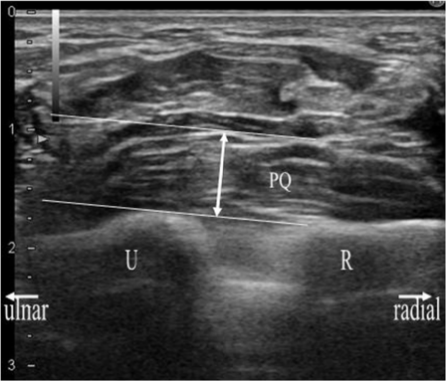
**Sonographic transverse image of the examined part of the distal forearm, including anatomical reference and measurements of PQ thickness, using a 17-MHz transducer.** Left hand of the same volunteer with Figure 2. We referred to the line tangential to the convex aspect of the ulna, which was drawn from the ulnar rim of the radius. PQ: pronator quadratus; R: radius; U: ulna; full-lined arrow: maximum thickness of PQ.

The sonographic appearance of the PQ was checked on both longitudinal and transverse images. When a probe is positioned on the volar side of the distal forearm, it allows for direct visualization of the flexor tendons and muscles, including the PQ, and the volar cortex of the radius in various planes. In the longitudinal plane, the PQ has a stippled, hypoechogenic, fusiform appearance. In the transverse plane, the two layers of the PQ (superficial and deep) are distinguished from one another by their alternating echogenicity [6]. The patients were seated with hand resting on table with elbow extended, forearm fully supinated, and wrist extended 5–10 degrees. The probe was positioned perpendicularly against the volar surface of the examined distal forearm with minimum pressure. On the longitudinal image, the probe was positioned along the flexor carpi radialis tendon (Figure 1). This image included a longitudinal section of the flexor carpi radialis tendon, PQ, volar cortex of the radius, and, on the distal end of the image, the distal margin of the bony prominence attached to the PQ (Figure 2). For the transverse image, we initially adopted the image of the unaffected hand in which the PQ showed maximal thickness when moving the probe proximally from carpus to forearm (Figures 3 and 4). Next, we adopted the image of the affected hand at the level located the same distance from the radiocarpal joint with that of the unaffected hand. Comparisons were made with the contralateral unaffected hand. Images were created on a display showing a side-by-side comparison between affected and unaffected hands. The PQ was considered to be swollen with disproportionate hyperechogenicity and/or thickening compared with the unaffected side at least in one of the two images.

Of the 55 patients, we advised 26 whose PQ was considered to be swollen to undergo MRI to check for occult bone injury. As a result, 25 patients comprising 15 children and 10 adults underwent MRI, excluding one patient who refused a further detailed study. All patients demonstrated right-hand dominance. Affected hands included 12 right hands and 13 left hands. Sonography and MRI were performed within 3 and 5 days following trauma (mean interval, 1.0 and 2.5 days), respectively. The interval between sonography and MRI was within 5 days (mean interval, 1.4 days). In the follow-up period, we also obtained radiographs every week until 3 weeks after the initial visit to evaluate whether delayed radiographic detection was possible if occult bone injury had been detected on MRI.

On each image, the maximal dorsal-volar thickness was measured to determine whether the PQ was thickened compared with the unaffected side. Considering the correspondence to the radiographic PQ sign, we measured the maximal thickness in millimeters on the volar side at the cortical level, which seemed to mainly represent the superficial layer of the PQ in the same manner as in the previous study of normal volunteers [7] (Figures 2 and 4). On the transverse image, we referred to a line tangent to the cortical bones of the radius and ulna (Figure 4).

All sonographic studies were performed using software from the Avius ultrasound system (Hitachi Medico Co., Japan). A linear array 13.5- or 17-MHz transducer (contact area, 14 × 59 mm) was used. All MRI studies were performed using a Signa Profile EXCITE (GE Yokogawa Medical System Co., Japan); the magnetic flux density was 0.2 tesla, with a series of sagittal and coronal images in proton density (PD)-weighted, short-term T1 inversion recovery (STIR) and T2*-weighted sequences. With regard to the assessment of bone lesions on MRI, the term “fracture” was used when a line of low intensity was present on both PD-weighted and T2*-weighted images. The term “bone bruising (contusion)” was used with the presence of bone marrow edema exhibiting ill-defined low intensity on PD-weighted images and higher intensity on both STIR and T2*-weighted images without a fracture line. One senior hand surgeon with 13 years of experience in surgery and 2 years of experience in ultrasound analyzed all images. We categorized the patients into three groups according to the type of occult bone fracture; radius fracture, radius bone bruising (contusion), and other wrist fracture. We also compared the PQ thickness between each two of these groups using the Student’s *t*-test. Results were deemed significant if *p* < 0.05.

## Results

**Figure 5:**
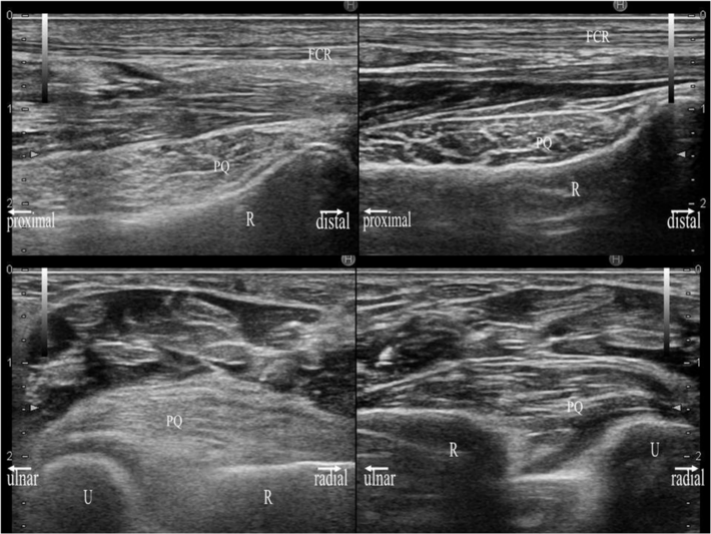
**Sonographic images of a 71-year-old man with a diagnosis of distal radius fracture of the left hand.** Sonographic images in the longitudinal (upper image) and transverse (lower image) planes were obtained using a 17-MHz transducer. Each image shows a side-by-side comparison of the right hand (right image) and left hand (left image). The PQ of the left hand is prominently thickened with hyperechogenicity. The PQ shows a convex appearance in either hand.

**Figure 6:**
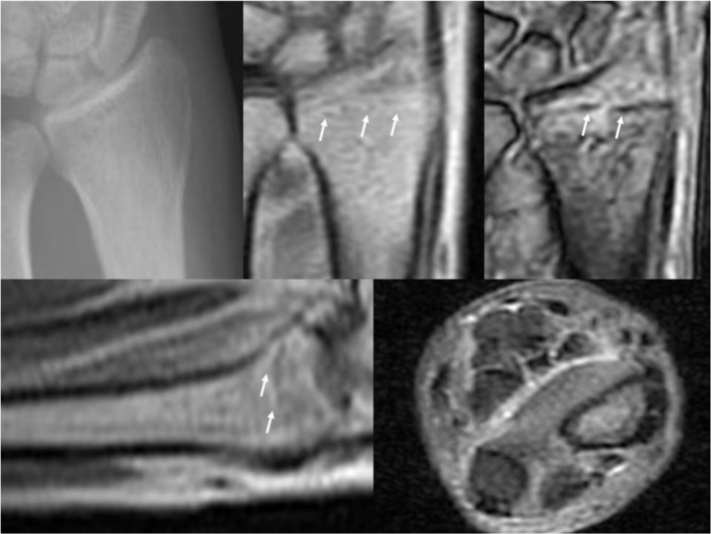
**Radiographic, and MRI images of the same patient with Figure** **5** **.** In upper image, a plain radiograph at the initial visit (left), a PD (middle) and a T2*-weighted (right) MRI coronal image are shown. In lower image, a PD-weighted MRI sagittal image (left) and STIR MRI transverse image (right) are shown. White arrows indicate the fracture line.

**Figure 7:**
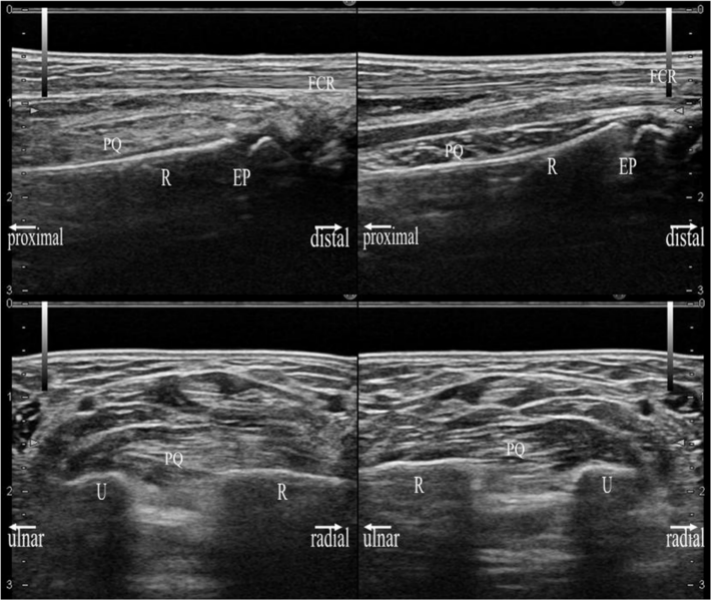
**Sonographic images of an 11-year-old girl with a diagnosis of distal radius contusion of the left hand.** Sonographic images in the longitudinal (upper image) and transverse (lower image) planes were obtained using a 17-MHz transducer. Each image shows a side-by-side comparison of the right hand (right image) and left hand (left image). The PQ of the left hand is locally thickened with hyperechogenicity on the radial side. The PQ shows a convex appearance in either hand. EP: epiphyseal plate.

**Figure 8:**
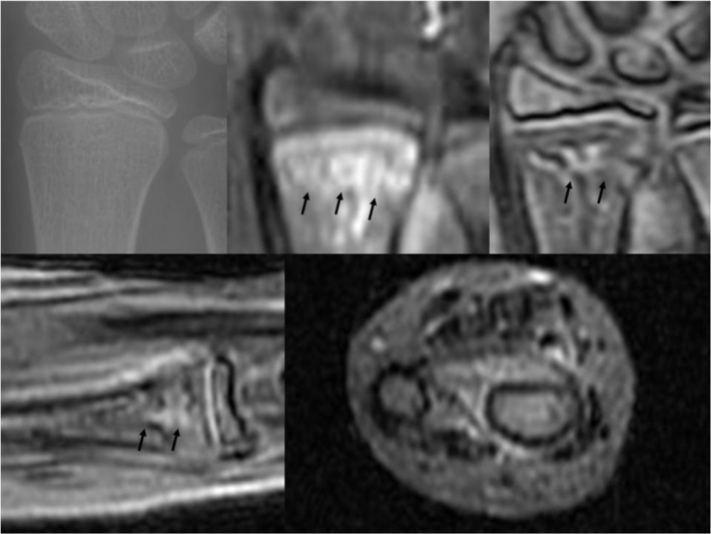
**Radiographic, and MRI images of the same patient with Figure** **7** **.** In upper image, a plain radiograph at the initial visit (left), a STIR (middle) and a T2*-weighted (right) MRI coronal image are shown. In lower image, a T2*-weighted MRI sagittal image (left) and STIR MRI transverse image (right) are shown. Black arrows indicate the bone bruising site.

**Figure 9:**
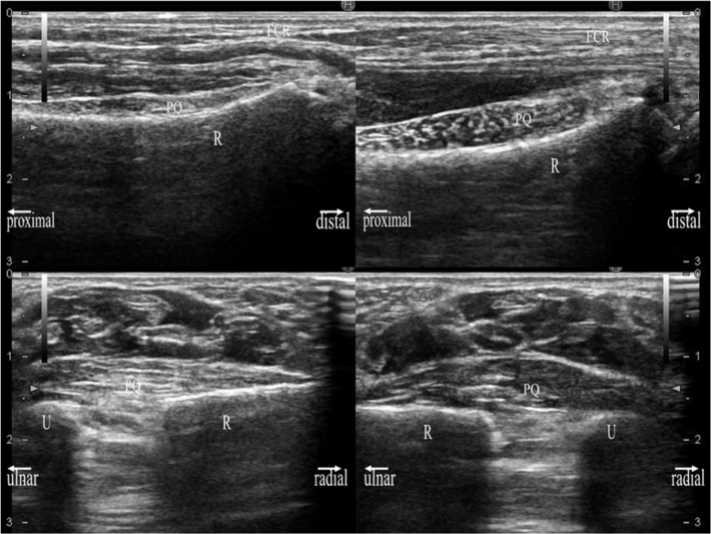
**Sonographic images of a 59-year-old woman with a diagnosis of wrist joint effusion of the right hand.** Sonographic images in the longitudinal (upper image) and transverse (lower image) planes were obtained using a 13.5-MHz transducer. Each image shows a side-by-side comparison of the right hand (right image) and left hand (left image). The PQ of the right hand is locally thickened. The PQ shows a convex appearance only in right hand.

**Figure 10:**
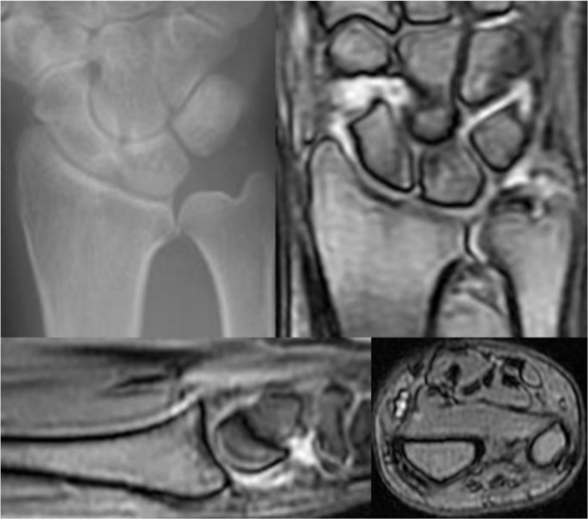
**Radiographic, and MRI images of the same patient with Figure** **9** **.** In upper image, a plain radiograph at the initial visit (left) and a T2*-weighted (right) MRI coronal image are shown. In lower image, T2*-weighted MRI sagittal (left) and transverse (right) images are shown.

Table 1 reports patients’ information regarding MRI and follow-up radiography. The patients underwent sonographic study and MRI comprised 32 men and 23 women [age, 9–80 years; mean, 26.3; standard deviation (SD), 22.8], and 15 men and 10 women [age, 10–80 years; mean, 32.64; SD, 24.8], respectively. Twenty-three patients (92.0%) had occult bone injury. Of these 23, the distal radius was the most frequent location for occult injury (detected in 20 patients; 9 [36.0%] with an occult fracture line (Figures 5 and 6) and 11 [44.0%] with bone bruising [contusion] (Figures 7 and 8). Two adult patients (8.0%) showed only wrist joint effusion on MRI (Figures 9 and 10). In children, all injuries detected in the distal radius were located on the proximal side of the epiphyseal line. Two patients (8.0%) had a solitary scaphoid injury, and one patient (4.0%) had a solitary ulna fracture. Four patients (16.0%) had two injured bones; they had occult radius injuries with scaphoid, capitate, or ulnar styloid injuries. In subsequent radiographs, bone injury was detected in 15 (78.9%) of 19 patients who underwent follow-up. We could not obtain the subsequent radiographs in four patients with occult bone injury because they did not visit our clinic within the follow-up period. The results of sonographic measurement of the PQ in involved patients are described in Table 2.

**Table 1:** **Patients’ information regarding MRI and follow-up radiography**

	**Period from trauma to sonography/MRI study**	**Final diagnosis on MRI**	**Follow-up radiography occult injury detected**
0 day	9/2	-	-
1 day	9/6	-	-
2 days	4/5	-	-
3 days	3/6	-	-
4 days	0/2	-	-
5 days	0/4	-	-
Distal radius contusion	-	10	-
Distal radius contusion/capitate fracture	-	1	-
Distal radius fracture	-	6	-
Distal radius fracture/Ulna styloid fracture	-	1	-
Distal radius fracture/Scaphoid contusion	-	1	-
Ulna fracture	-	1	-
Scaphoid fracture	-	2	-
Scaphoid fracture	-	1	-
***Joint effusion***	-	2	-
Did not undergo follow-up radiography	-	-	4
1 week	-	-	3
2 weeks	-	-	2
3 weeks	-	-	10
No detected	-	-	4

The numbers of patients are described in the box.

Bold and italics indicate final diagnoses other than occult bone injury.

**Table 2:** **Sonographic information of PQ, and their statistical analyses**

**Sonographic measurement of PQ thickness (mm)**					
**Longitudinal image**			**Transverse image**		
**A**	**UA**	**Difference** ^**†**^	**A**	**UA**	**Difference** ^**†**^
6.2 ± 1.5	4.5 ± 1.2	1.7 ± 1.1	7.6 ± 2.0	5.5 ± 1.1	2.0 ± 1.7
(3.7 – 9.6)	(2.3 – 6.7)	(0.1 – 5.0)	(4.6 – 13.2)	(3.6 – 7.5)	(0.3 – 6.8)

PQ thicknesses are given as mean value ± standard deviation with ranges in parentheses.

A: affected hand.

UA: unaffected hand.

^†^Difference in PQ thickness between affected and unaffected hands.

There was no significant difference in PQ thickness between any two groups of occult bone fracture (radius fracture vs. radius bone bruising [contusion], *p* = 0.58 in longitudinal image and *p* = 0.52 in transverse image; radius fracture vs. other wrist fracture, *p* = 0.86 in longitudinal image and *p* = 0.68 in transverse image; radius bone bruising [contusion] vs. other wrist fracture, *p* = 0.82 in longitudinal image and *p* = 0.90 in transverse image).

## Discussion

The PQ is reportedly a key soft tissue in the diagnosis of radiographically undetectable fractures [1,2,4,8,9]. It is attached to the anterior aspects of the distal one-sixth of the radius and ulna and the distal radioulnar joint (DRUJ). The PQ comprises two layers: the superficial and deep layers [10,11]. Johnson and Shrewsbury [10] reported that the superficial layer was longer and wider but thinner than the deep layer, with average thicknesses of 0.2 cm for the superficial layer and 0.4 cm for the deep layer based on the dissection results of 12 forearms cross-sections made at one centimeter intervals. The superficial layer is considered to act as a prime contributor to pronation of the forearm in coordination with the pronator teres muscle [10-12]. The deep layer is active during both pronation and supination [12] and takes part in stabilizing the DRUJ by inserting onto the joint capsule [10-12]. With regard to the wrist, a 62.4% sensitivity of occult bone injuries on MRI was reported in the patients with negative radiographs and persistent pain following trauma [13], and a 40% to 65% sensitivity of scaphoid fracture on MRI was reported in the clinically suspicious patients with a normal series of plain radiographs [14,15].

The disarranged fat stripe of the PQ on radiographs (the PQ sign) is considered to be a highly predictive but low-sensitivity finding of subtle bone fractures, particularly of the distal radius and ulna [3,8], whereas Zammit-Maempel et al. [4] reported only 51% of forearm fractures showed this sign. Annamalai and Raby [5] reported that this sign was a poor predictor of occult fractures detected on MRI in their retrospective study. Ultrasonography has distinct merits in terms of noninvasiveness and time- and cost-effectiveness, and it is best used when there is a specific clinical question regarding a well-localized abnormality [16]. Although ultrasonography is not able to make a quantitative diagnosis of bone marrow, sonographic swelling of the PQ predicted occult bone injury with high probability of 92%. Moreover, sonographic swelling of the PQ might support a recommendation for further detailed imaging studies such as MRI for the patients with normal radiographs. With regards to the thickness of the PQ, Zammit-Maempel et al. [4] measured the distance of the normal pronator fat stripe from the radius on lateral radiographs of the patients who presented with acute wrist trauma. They reported that the distance had a very wide variation with a mean value of 4.97 mm (5.72 mm in males and 4.22 mm in females) in 773 normal patients without abnormal soft tissue and/or bone injury, and that the patients with fractures of the distal radius and ulna, and distal radius had a significantly greater distance with mean values of 6.98 mm and 6.51 mm, respectively (*p* < 0.01). Sasaki et al. [8] reported the distance was less than 7 mm in 92% of 72 normal control subjects in which pronator fat shadow could be detected, and over 7 mm in 93% of 29 recent distal radius fractures under the same condition. Our results of sonographic measurement in normal subjects (unaffected hand) showed mean values of 4.5 mm in longitudinal, and 5.5 mm in transverse image.

A consensus has not been reached regarding whether occult bone injuries of the wrist should be immobilized and how long they should be immobilized. However, it is important to precisely determine the cause of symptoms and to inform patients. Actually, all four occult bone injuries in our study were contusions of the radius or scaphoid in children and could not be detected on subsequent radiographs. Even if a physician continues to check the radiographs carefully, a substantial number of occult bone injuries seem to be overlooked.

Our study has several limitations. First, our results can show only positive predictive value. We did not check the MRI of the patients with a normal sonographic appearance of the PQ. Despite economic and ethical problems, a closer study of such cases is desirable to investigate the accuracy of sonographic PQ swelling in terms of occult injury of the wrist. Second, this study might contain substantial bias in the selection of patients undergoing sonography because pain disproportionate to normal radiographs, the requirement for sonography examination, is quite examiner-subjective. We may have overlooked some patients with a swollen PQ. Third, the decision of sonographic PQ swelling depended on the comparison of the PQ thickness between affected and unaffected hands. Previous sonographic study revealed that atrophy of the PQ may be present in asymptomatic patient, and that loss in bulk of the PQ may be isolated and not related to anterior osseous neuropathy [17]. Although the authors described that it is not possible to identify or suppose a definite reason for this phenomenon, they speculated that the generic, occupational factors and anatomical variants in the innervation might be responsible. It is undeniable that atrophic PQ in the unaffected hand might be decided to be normal in our study.

## Conclusions

In conclusion, sonographic swelling of the PQ might be indicative of occult bone injury in patients with normal radiographs following acute trauma. Ultrasonography might be a convenient adjunct for the decision of further detailed imaging studies in these patients as a noninvasive and portable tool that can be performed in the clinic and emergency room.
